# Mechanistic Insights into Pancreatic Lipase Inhibition by Pea-Derived Peptides: Integrating Process Optimization, Activity Assays, Docking, and Molecular Dynamics

**DOI:** 10.3390/foods15091523

**Published:** 2026-04-28

**Authors:** Yi Zhao, Jinhong Wang, Xiang Li, Guizhao Liang

**Affiliations:** Key Laboratory of Biorheological Science and Technology, Ministry of Education, Bioengineering College, Chongqing University, Chongqing 400044, China

**Keywords:** pancreatic lipase, inhibitory peptides, molecular dynamics simulations, MM/PBSA, structure–activity relationship

## Abstract

Pancreatic lipase (PL) plays a central role in dietary lipid digestion and is a promising target for food-derived inhibitors. In this study, pea protein hydrolysates (PPHs) with PL inhibitory activity were prepared by enzymatic hydrolysis and characterized for their functional and peptidomic properties. Compared with pea protein isolate, PPH showed lower surface hydrophobicity, and moderate antioxidant activity. Peptidomic analysis identified 1740 peptides in the active hydrolysate. Combined in silico screening and in vitro validation further identified three peptides, GFSL, WFE, and FGF, as effective PL inhibitors, with IC_50_ values of 337.81 ± 17.32, 473.32 ± 19.61, and 689.45 ± 39.32 μM, respectively. Molecular simulations indicated that these peptides interact with the catalytic pocket of PL mainly through hydrophobic interactions, van der Waals forces, and hydrogen bonding, with Ile79 serving as a key residue for peptide recognition. Overall, these findings indicate the potential of pea-derived peptides as natural PL inhibitors and support their application as functional food ingredients for modulating lipid digestion.

## 1. Introduction

In recent years, the global prevalence of obesity has continued to rise, making it one of the most pressing challenges in global public health [1]. Obesity is not only a significant risk factor for various non-communicable diseases, such as type 2 diabetes, cardiovascular diseases, and dyslipidemia, but it is also closely associated with the occurrence of gastrointestinal cancers [2] and contributes substantially to the global burden of premature death [3]. Studies have shown that a weight loss of over 10% can significantly improve obesity-related complications, including type 2 diabetes mellitus, hypertension, and non-alcoholic fatty liver disease, while also significantly enhancing the quality of life of patients [4]. Therefore, developing safe, effective, and long-term weight management strategies is of great importance for reducing the burden of obesity and its related diseases, improving population metabolic health outcomes, and advancing public health levels.

Pancreatic lipase (PL) is a key enzyme secreted by the pancreas that plays a central role in the digestion of dietary fats, primarily responsible for hydrolyzing triglycerides into free fatty acids and glycerol, thereby promoting lipid absorption. Therefore, inhibiting PL activity to reduce fat absorption is considered an effective strategy for weight management [5]. Although orlistat is an approved PL inhibitor with demonstrated clinical efficacy, its long-term use is often associated with gastrointestinal discomfort and reduced absorption of fat-soluble nutrients [6]. Therefore, the search for natural PL inhibitors with improved safety and tolerability has attracted increasing attention.

In recent years, food-derived proteins and their bioactive peptides have gained increasing attention in obesity management due to their high safety, good biocompatibility, and edibility [7]. Enzymatic hydrolysis can release short peptides from natural proteins, and these peptides may inhibit PL by interacting with residues in or near the catalytic pocket, thereby interfering with substrate recognition or catalysis [8]. Compared with synthetic inhibitors, naturally derived peptides generally exhibit lower toxicity and better suitability for food and nutritional applications [7]. Among various plant protein sources, pea protein has attracted considerable attention due to its high protein content, balanced essential amino acid composition, and low allergenicity [9]. Previous studies have shown that pea protein hydrolysates (PPHs) possess diverse bioactivities, including antioxidant activity [9]. Additionally, pea protein has been extensively applied in the industry for the encapsulation of bioactive compounds and the fabrication of emulsion-based delivery systems [10,11,12]. Its favorable nutritional profile and functional characteristics make it an attractive protein source for the generation of bioactive hydrolysates and peptides [13]. In this context, antioxidant activity is also of interest, since it may enhance oxidative stability during processing and storage and provide added health-promoting potential [14].

In the present study, PPHs with PL-inhibitory activity were prepared by enzymatic hydrolysis, and the active hydrolysate was characterized in terms of its physicochemical properties and peptide composition. The antioxidant capacity of the hydrolysate was also evaluated using 2,2-diphenyl-1-picrylhydrazyl radical (DPPH·) and 2,2′-azinobis-(3-ethylbenzothiazoline-6-sulfonic acid) radical cation (ABTS^+^·) scavenging assays. Liquid chromatography-tandem mass spectrometry (LC-MS/MS)-based peptidomic profiling was combined with bioactivity prediction, toxicity evaluation, molecular docking, and in vitro validation to identify key PL-inhibitory peptides. Furthermore, molecular dynamics (MD) simulations, Molecular Mechanics/Poisson–Boltzmann Surface Area (MM/PBSA) calculations, and residue-level energy decomposition were employed to elucidate the molecular interactions between the identified peptides and PL. This study provides structural and mechanistic insight into the inhibition of PL by pea-derived peptides and contributes to the molecular-level understanding of food-derived protein-based enzyme inhibitors.

## 2. Materials and Methods

### 2.1. Materials and Reagents

PL (Type II, L3126) was purchased from Sigma-Aldrich (St. Louis, MO, USA). p-nitrophenyl butyrate (pNPB), pea protein isolate (PPI), trypsin, pepsin, papain, alkaline protease, and neutral protease were purchased from Shanghai YuanYe Biotechnology Co., Ltd. Peptides were synthesized by Nanjing JieTai Biotechnology Co., Ltd. (Nanjing, China), with a purity greater than 95%. All other chemicals and reagents used in this study were of analytical grade.

### 2.2. Optimization of PPI Enzymatic Hydrolysis Process

Enzymatic hydrolysis of 2% (*w*/*v*) PPI solution was performed using five commonly used proteases in food protein hydrolysis, namely trypsin, pepsin, papain, alkaline protease, and neutral protease [15,16], to compare their hydrolytic effects on PPI. These enzymes were selected because they differ in substrate specificity and hydrolytic behavior, allowing a comprehensive screening of suitable proteases for PPI hydrolysis. Since each protease has its own optimal catalytic pH and temperature, hydrolysis was conducted under the respective optimal conditions for each enzyme, as summarized in Table S1, to ensure sufficient enzymatic activity and enable a meaningful comparison of hydrolysis efficiency. After the enzymatic reaction, the reaction mixture was heated in a 90 °C water bath for 15 min to inactivate the enzyme. The mixture was then centrifuged at 4000× *g* for 10 min, and the supernatant was collected and freeze-dried. The freeze-dried hydrolysate was re-dissolved to an initial concentration of 5 mg/mL for subsequent activity assays. Using pH 10, temperature 50 °C, hydrolysis time of 3 h, and enzyme-to-substrate ratio (E/S) of 5000 U/g as the baseline conditions, a preliminary optimization of the enzymatic hydrolysis process was conducted through single-factor experiments. Response surface methodology (RSM) was applied using the RSM package [17], and the PL inhibition rate was selected as the response variable. A four-factor optimization model was constructed using central composite design (CCD), with a total of 30 experimental points (Table S2).

### 2.3. PL Inhibition Assay

Tris-HCl buffer (pH = 7.5) was used as the solvent. A 5 mg/mL solution of PL was centrifuged at 10,000× *g* for 5 min, and the supernatant was collected as the PL stock solution, which was stored under appropriate conditions for later use. A certain amount of PL stock solution was mixed with inhibitor solutions at gradient concentrations, and incubated at 37 °C for 20 min. After incubation, pNPB was added to the reaction system to a final concentration of 0.5 mg/mL, and the reaction was continued at 37 °C for an additional 10 min. After the reaction, the absorbance of each reaction system was measured at 405 nm.

(1) $$PL\;inhibition\;rate\;\left(\%\right)=\frac{\left[\left(A-B\right)-\left(C-D\right)\right]}{\left(A-B\right)}\times 100\%$$

where *A* represents the absorbance of the control group containing PL, but no inhibitor, with an equal volume of Tris-HCl buffer replacing the inhibitor. *B* represents the absorbance of the blank control group containing neither PL nor inhibitor, both replaced by buffer. *C* represents the absorbance of the sample group containing both PL and inhibitor. *D* represents the absorbance of the sample blank group containing inhibitor, but no PL, with an equal volume of buffer replacing the enzyme.

### 2.4. Surface Hydrophobicity

The surface hydrophobicity of PPI and PPH was determined using 8-anilino-1-naphthalenesulfonic acid (ANS) as a fluorescent probe. PPI and PPH were dispersed in deionized water to final concentrations of 0.05, 0.10, 0.15, 0.20, and 0.25 mg/mL. For each measurement, 4 mL of sample solution was mixed with 20 μL of 8 mM ANS solution and incubated in the dark for 15 min. Fluorescence intensity was then measured using a fluorescence spectrophotometer at an excitation wavelength of 390 nm and an emission wavelength of 470 nm. The surface hydrophobicity index (H_0_) was calculated as the initial slope of the linear regression of fluorescence intensity versus protein concentration.

### 2.5. Antioxidant Activity

PPH antioxidant activity was evaluated using ABTS^+^· and DPPH· radical scavenging assays. The ABTS^+^· working solution was prepared by reacting ABTS solution with potassium persulfate in the dark, followed by dilution with phosphate-buffered saline (PBS) to an absorbance of 0.70 ± 0.02 at 734 nm. The DPPH· working solution was prepared in 95% ethanol. PPH samples at different concentrations were mixed with either ABTS^+^· or DPPH· working solution and incubated in the dark, after which the absorbance was measured at 734 and 517 nm, respectively. Blank, control, and test wells were included to correct for interference from the intrinsic color and turbidity of the samples. Radical scavenging activity was calculated according to the following equation.

(2) $$Radical\;scavenging\;rate\;\left(\%\right)=(1-\frac{A_{test}-A_{control}}{A_{blank}})\times 100\%$$

where *A_test_* is the absorbance of the reaction mixture containing both sample and radical solution, *A_control_* is the absorbance of the sample background, and *A_blank_* is the absorbance of the blank.

### 2.6. LC-MS/MS Peptide Identification

Five mg of PPH was used for sample preparation with SDS–DTT–Tris lysis buffer (4% SDS, 100 mM Tris-HCl, pH 7.6, 0.1 M DTT), followed by ultrasonic treatment, boiling water bath, and centrifugation to collect the supernatant. The proteins were separated using an RP-C18 column with solvent A (0.1% formic acid aqueous solution) and solvent B (0.1% formic acid acetonitrile solution). Mass spectrometry was performed using a Q Exactive HF-X mass spectrometer in positive ion mode for 60 min, collecting 10 MS2 fragment spectra after each full scan. The mass spectrometry data were analyzed by MaxQuant 1.5.5.1 software for database search, and the peptide identification and quantification results were obtained. The mass spectrometry proteomics data have been deposited to the ProteomeXchange Consortium (https://proteomecentral.proteomexchange.org, accessed on 25 March 2026) via the iProX partner repository [18,19] with the dataset identifier PXD076130.

### 2.7. Virtual Screening

Peptides were predicted using online tools PeptideRanker (https://peptide.ucd.ie/peptideranker/) and ToxinPred 3.0 [20], selecting peptides with high activity and no toxicity risk as candidate molecules. The crystal structure of PL (PDB ID: 1ETH) was used as the receptor for molecular docking, and only chain A was retained for docking analysis. The receptor was preprocessed using AutoDockTools (version 1.5.6) by removing crystallographic water molecules and the co-crystallized ligand, followed by the addition of hydrogen atoms and assignment of charges. The three-dimensional structures of the peptides were constructed using the Yinfu Technology Cloud Platform (https://cloud.yinfotek.com/), energy-minimized using Open Babel under the MMFF94 force field with the steepest descent algorithm, and then converted into PDBQT format using AutoDockTools (version 1.5.6). The prepared receptor structure was saved in PDBQT format. Molecular docking was performed using AutoDock Vina 1.2.0 [21]. in blind docking mode. The docking search space was defined by a grid box centered at (62.79, 29.33, 118.45), with dimensions of 74.42 × 58.75 × 78.33 Å. The exhaustiveness parameter was set to 32. A total of 10 poses were generated for each docking run, and the pose with the lowest docking score was selected for subsequent MD simulations. The binding sites and interaction types of the peptide-enzyme complexes were visualized using PyMOL 2.6.0 [22] and PLIP [23].

### 2.8. MD Simulation

MD simulations were performed using GPU-accelerated GROningen MAchine for Chemical Simulations (GROMACS). The optimal docking pose of each peptide–PL complex was used as the initial structure. The protein was parameterized using the AMBER99SB-ILDN force field, and the protonation states of the protein and peptides were assigned according to the default ionization states of standard amino acid residues under this force field. Since all peptide residues were standard amino acids, the peptide topology and parameters were generated automatically using the pdb2gmx module in GROMACS under the same force field. Each system was solvated in a cubic box using the TIP3P water model, with a minimum distance of 1.0 nm between the solute and the box edge. Na^+^ or Cl^−^ ions were added to neutralize the net charge of the system. Energy minimization was performed using the steepest descent algorithm for up to 50,000 steps until the maximum force was below 1000 kJ mol^−1^ nm^−1^. Subsequently, NVT equilibration was carried out for 100 ps at 300 K using the V-rescale thermostat with position restraints, followed by NPT equilibration for 100 ps at 300 K and 1 bar using the Parrinello–Rahman barostat, also with position restraints. After equilibration, a 100 ns production MD simulation was performed with a time step of 2 fs under periodic boundary conditions. Long-range electrostatic interactions were treated using the particle mesh Ewald (PME) method, while the short-range Coulomb and van der Waals cutoffs were both set to 1.0 nm. Bonds involving hydrogen atoms were constrained using the LINCS algorithm. Trajectory frames were saved every 10 ps. The trajectory files (.dcd) were processed and pre-processed using Visual Molecular Dynamics (VMD) 1.9.3 [24]. Dynamic cross-correlation matrix (DCCM) analysis was performed using Bio3D [25] based on Cα atom trajectories, and principal component analysis (PCA) was carried out using the 90–100 ns trajectory segment to characterize the dominant conformational changes during the simulations.

### 2.9. Interaction Analysis and Energy Decomposition

The 90–100 ns segment of the MD trajectories was selected because the root mean square deviation (RMSD) profiles had stabilized during this period. MM/PBSA and per-residue free-energy decomposition analyses were performed using gmx_MMPBSA [26]. Since trajectory frames were saved every 10 ps, 1000 snapshots extracted from the 90–100 ns segment were used for the calculations. ProLIF [27] was used for interaction analysis. The total binding free energy (Δ*G_bind_*) of the receptor-ligand complex was calculated using the formula shown in Equation (2). Additionally, residue–residue contact score (RRCS) analysis [28] was performed to detect subtle conformational dynamics during the MD simulations and to analyze the residue-peptide interaction changes during the complex binding process.

(3) $$\Delta G_{bind}=\Delta G_{ele}+\Delta G_{vdW}+\Delta G_{polar}+\Delta G_{nonpolar}$$

where Δ*G_ele_* is the electrostatic interaction energy between the ligand and the receptor, Δ*G_vdW_* is the van der Waals interaction energy, and Δ*G_polar_* and Δ*G_nonpolar_* describe the polar and nonpolar components of the solvation contribution during binding.

### 2.10. Data Analysis

Each experiment was repeated at least 3 times independently. The experimental results were statistically analyzed using R 4.3.1 software [29] and Excel software (2021). Data are expressed as mean ± standard deviation (mean ± SD).

## 3. Results and Discussion

### 3.1. Process Optimization for the Production of PPH with PL Inhibitory Activity

**Figure 1:**
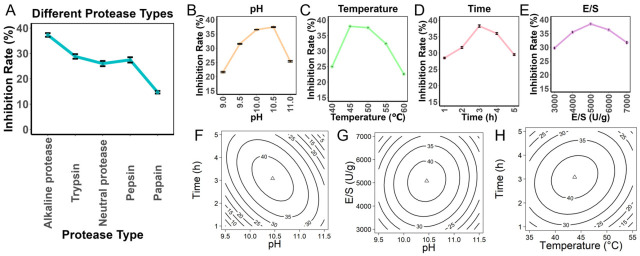
Effects of hydrolysis conditions on PL inhibitory activity: (**A**) Effect of protease type. (**B**) Effect of pH. (**C**) Effect of temperature. (**D**) Effect of hydrolysis time. (**E**) Effect of enzyme-to-substrate ratio (E/S). (**F**–**H**) Response surface plots showing the effects of key hydrolysis variables on PL inhibitory activity.

In this study, five different proteases (trypsin, pepsin, papain, alkaline protease, and neutral protease) were selected for the enzymatic hydrolysis of PPI. The results showed significant differences in the PL inhibitory activity of the resulting products, highlighting the critical impact of enzyme cleavage specificity on product bioactivity. Specifically, alkaline protease exhibited the highest inhibitory activity (Figure 1A), so alkaline protease was chosen for subsequent optimization studies. The single-factor experimental results indicated that the highest inhibition rate was achieved at pH 10.5 (Figure 1B), with the optimal reaction temperature at 45 °C (Figure 1C), the highest inhibition rate was obtained after 3 h (Figure 1D), and the best inhibition effect at an E/S of 5000 U/g (Figure 1E). Based on these findings, the preliminary optimized conditions were determined to be pH 10.5, temperature 45 °C, hydrolysis time 3 h, and E/S 5000 U/g.

To precisely analyze the interactions of multiple factors, a response surface model was constructed using CCD. The factors and response values used in the experiment are shown in Table S2. The model in terms of coded variables was: Inhibition = 42.2959 − 0.5914A − 1.6826B + 0.4634C + 0.4912D − 0.0466A × B − 3.2060A × C + 0.6791A × D + 1.0844B × C − 0.2064B × D + 0.5422C × D − 4.3974A^2^ − 3.2063B^2^ − 3.2047C^2^ − 3.2439D^2^ where (A: pH, B: Temperature, C: Time, D: E/S). Statistical validation showed that the model was highly significant (*p* < 0.001), with no significant lack of fit (*p* = 0.7073). The goodness of fit (R^2^ = 0.9867) and predictive ability (adjusted R^2^ = 0.9744) were excellent, and the analysis of variance (ANOVA) results are shown in Table S3. Further analysis revealed significant interactions between pH and hydrolysis time (*p* < 0.05), indicating that the optimal enzyme activity range is influenced not only by individual pH and time but also by their interaction (Figure 1F). Similarly, a significant interaction was observed between pH and E/S (Figure 1G), suggesting that the hydrolysis outcome was influenced by both the individual effects of pH and E/S and their interaction. Under different pH conditions, the E/S affects the hydrolysis process differently, thereby affecting the properties and bioactivity of the resulting hydrolysates. Therefore, rational adjustment of pH and E/S combinations can effectively improve enzymatic hydrolysis efficiency. Significant interaction was also observed between hydrolysis time and temperature (Figure 1H), with the contour lines showing a dense elliptical shape, while no significant interaction was found between other factors (*p* > 0.05). Ultimately, the optimal conditions predicted by the model were: pH 10.45, temperature 43.75 °C, hydrolysis time 3.08 h, and E/S 5081.14 U/g. The experimental results under the optimized conditions showed an inhibition rate of 41.76% ± 2.5%, with a deviation of only 0.81% from the predicted value of 42.57%, further validating the accuracy of the model. Therefore, RSM proved to be effective for optimizing the hydrolysis conditions for the production of PPH with PL inhibitory activity.

### 3.2. The Surface Hydrophobicity of PPH Is Lower than That of PPI

**Figure 2:**
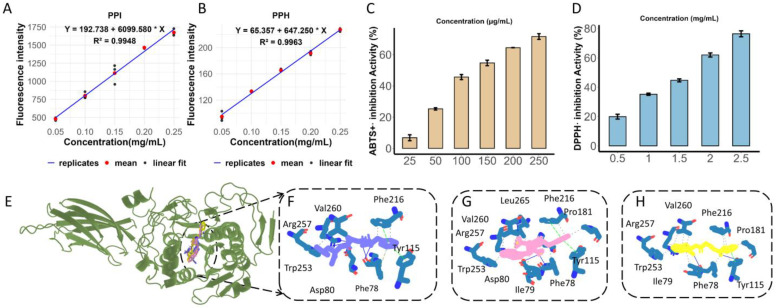
Physicochemical properties, antioxidant activity, and molecular docking analysis of PPH: (**A**) ANS fluorescence of PPI at different concentrations for surface hydrophobicity determination. (**B**) ANS fluorescence of PPH at different concentrations for surface hydrophobicity determination. (**C**) ABTS^+^· radical scavenging activity of PPH. (**D**) DPPH· radical scavenging activity of PPH. (**E**) Binding site of peptides in PL. (**F**–**H**) Docking poses of GFSL (blue), WFE (pink), and FGF (yellow) in the active pocket of PL.

The relationships between fluorescence intensity and protein concentration for PPI and PPH are shown in Figure 2A and Figure 2B, respectively, with good linear fitting (R^2^ > 0.99). The surface hydrophobicity index (H_0_), represented by the slope of the fitted line, was markedly higher for PPI (6099.580) than for PPH (647.250), indicating that enzymatic hydrolysis reduced the surface hydrophobicity of pea protein. This result suggests that PPI possessed more exposed hydrophobic groups on its molecular surface, whereas hydrolysis decreased the exposure of hydrophobic regions and increased the affinity of the hydrolysate for the aqueous phase. Such changes in surface properties may be associated with the structural rearrangement and peptide fragmentation induced by enzymatic treatment, and could influence the functional behavior of the hydrolysate in food systems.

### 3.3. ABTS^+^· and DPPH· Radical Scavenging Activities of PPH

As shown in Figure 2C,D, PPH exhibited concentration-dependent scavenging activities against both ABTS^+^· and DPPH· radicals, with consistently higher activity toward ABTS^+^· than DPPH·. In the ABTS^+^· assay, the scavenging rate increased markedly with increasing concentration, whereas in the DPPH· assay, it increased from approximately 20% to 77% over the concentration range of 0.5–2.5 mg/mL. These results suggest that PPH may possess radical-quenching capacity through hydrogen atom transfer and/or electron transfer mechanisms. The higher scavenging activity observed in the ABTS^+^· assay may reflect differences in radical reactivity and steric accessibility between the two assay systems. In addition, the reduced surface hydrophobicity of PPH after hydrolysis may facilitate the exposure of reactive groups, while the generated small peptides may improve diffusion and reaction accessibility. Taken together, these results indicate that PPH possesses appreciable in vitro antioxidant activity, particularly in the ABTS^+^· system, and may serve as a multifunctional ingredient when considered together with its PL inhibitory activity.

### 3.4. Integrated Screening and Activity-Based Identification of PL-Inhibitory Peptides

A total of 1740 peptide sequences were identified from the PPH. Given the advantages of ultrashort peptides, including ease of synthesis, good tissue penetration, and low immunogenicity, this study focused on screening peptides with a length of no more than 5 amino acids for further investigation (Table S4). These peptides are designated according to their amino acid sequences using the one-letter code. Before molecular docking and activity validation, potential high-activity, low-toxicity peptides were screened using PeptideRanker and ToxinPred 3.0. The selected candidate peptides were then subjected to molecular docking analysis with PL using AutoDock Vina software, yielding five candidate peptides: LFL, LFE, GFSL, WFE, and FGF.

The PL inhibition assay results showed that GFSL, WFE, and FGF had IC_50_ values of 337.81 ± 17.32 μM, 473.32 ± 19.61 μM, and 689.45 ± 39.32 μM, respectively, while LFL and LFE exhibited weaker inhibition of PL activity, with IC_50_ values greater than 1 mM (Table 1). Although WFE and FGF showed more favorable Vina scores than GFSL, GFSL exhibited the strongest in vitro PL inhibition, indicating that docking scores alone were insufficient to predict inhibitory potency. Therefore, GFSL, WFE, and FGF were selected for further investigation, providing a basis for subsequent structure-activity relationship analysis. The docking interaction sites of the protein-peptide complexes are shown in Figure 2E. GFSL was predicted to form a hydrogen bond with Phe78, π–π stacking interactions with Phe216 and Tyr115, and hydrophobic interactions with Val260, Arg257, Trp253, and Asp80 (Figure 2F). WFE can form hydrogen bonds with Asp80 and Phe78, π–π stacking interactions with Phe216 and Tyr115, and hydrophobic interactions with Pro181, Phe216, Val260, Arg257, Trp253, and Leu265 (Figure 2G). FGF can form hydrogen bonds with Phe78 and Tyr115, and hydrophobic interactions with Ile79, Tyr115, Trp253, Arg257, and Val260 (Figure 2H). These results indicate that active peptides can favorably bind to key amino acid residues surrounding the PL active pocket through hydrogen bonding, π–π stacking, and hydrophobic interactions, which may contribute to their inhibitory activity against PL.

### 3.5. MD Simulations Reveal the Dynamic Behavior and Binding Characteristics of Peptide–PL Complexes

**Figure 3:**
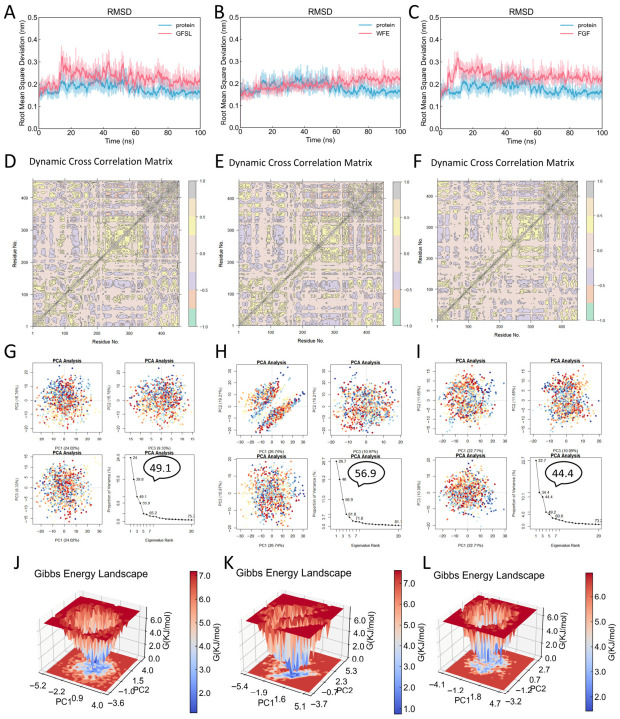
Molecular dynamics analysis of peptide–PL complexes: (**A**–**C**) RMSD profiles of the GFSL–PL, WFE–PL, and FGF–PL complexes during simulation. (**D**–**F**) DCCM of PL in the GFSL–PL, WFE–PL, and FGF–PL complexes (grey, correlated motions; cyan, anti-correlated motions). (**G**–**I**) PCA of the essential motions of PL in the GFSL–PL, WFE–PL, and FGF–PL complexes. (**J**–**L**) FEL constructed from PC1 and PC2 of the GFSL–PL, WFE–PL, and FGF–PL complexes.

We performed MD simulations to analyze the dynamic behavior of three peptide–PL complexes (GFSL–PL, WFE–PL, and FGF–PL) over 100 ns. The RMSD of the three peptide–PL complexes remained within a small fluctuation range during the equilibrium phase (Figure 3A–C), indicating that all three peptide–PL complexes showed limited RMSD fluctuations during the simulations without obvious structural destabilization. The Dynamical Cross-Correlation Matrix (DCCM) results showed that GFSL, WFE, and FGF all displayed cooperative motions among the surrounding residues in the PL binding pocket, suggesting that these coordinated motions may be associated with the stability of peptide binding to PL (Figure 3D–F). This further revealed the complexity and dynamic nature of peptide–PL interactions. Principal Component Analysis (PCA) identified the major motion modes of the complexes (Figure 3G–I), with the first three principal components (PC1–PC3) explaining over 40% of the overall movement, indicating that the major conformational changes in the system can be described by a few dominant motion modes. Based on this, to select representative stable conformations from the MD simulation and further analyze the peptide–PL binding mode, we constructed free energy landscapes (FEL) using PC1 and PC2 as reaction coordinates. The results showed that the GFSL (Figure 3J), WFE (Figure 3K), and FGF (Figure 3L) complexes each exhibited at least one distinct low-energy well in their energy distributions, indicating that all three systems sampled one or more low-energy conformational states during the later stage of the simulations, providing a reliable structural foundation for identifying key binding sites and interaction mechanisms in subsequent analyses.

Taken together, based on the combined analyses of RMSD, DCCM, PCA, and FEL, GFSL, WFE, and FGF were all found to be capable of binding to PL and forming favorable interactions with the protein. The differences in their inhibitory activities are not due to an increase in overall conformational fluctuations, but rather likely related to their binding modes and their ability to interact with key residues in the PL binding pocket. Therefore, these results not only support the binding behavior of the peptide–PL complexes but also provide reliable structural and dynamic insights for the precise identification of key binding sites and the elucidation of their inhibitory mechanisms.

### 3.6. MM/PBSA and RRCS Analysis Reveal Binding Energies and Key Sites of Peptide–PL Interactions

To further evaluate the binding stability of the GFSL–PL, WFE–PL, and FGF–PL complexes during the simulations, the binding free energies of the three peptide–PL complexes were calculated using the MM/PBSA method. The results showed that the binding free energies for the GFSL–PL, WFE–PL, and FGF–PL complexes were −26.24 ± 5.25 kcal/mol, −22.92 ± 3.43 kcal/mol, and −17.37 ± 3.20 kcal/mol, respectively. The MM/PBSA results showed the same overall ranking trend as the experimental IC_50_ values, with GFSL exhibiting the most favorable binding free energy and the strongest inhibitory activity in vitro. Overall, the MM/PBSA results provided supportive computational evidence for the experimental inhibitory activities among the three peptides.

**Figure 4:**
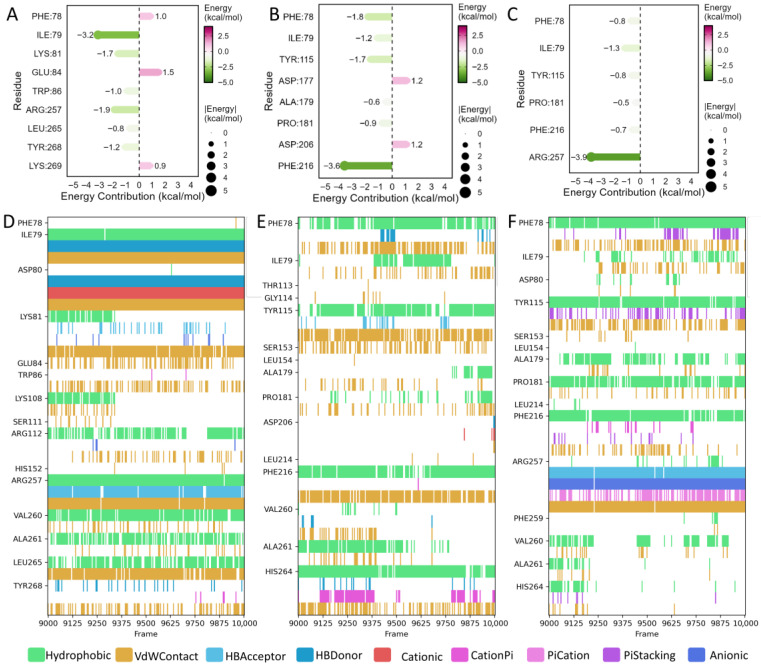
MM/PBSA energy decomposition and interaction fingerprint analysis of peptide–PL complexes: (**A**–**C**) Residue-wise MM/PBSA energy contributions in PL for the GFSL–PL, WFE–PL, and FGF–PL complexes. (**D**–**F**) Interaction fingerprints between PL and GFSL, WFE, and FGF.

Residue-level energy decomposition was further performed to identify residues that stabilize peptide binding. Residues with an absolute contribution of ≥0.5 kcal/mol to the binding free energy were defined as key binding hotspots. In the GFSL–PL complex, Ile79, Lys81, Trp86, Arg257, Leu265, and Tyr268 made pronounced favorable contributions to binding (Figure 4A). Analysis of interaction types between residues and the peptide indicated that GFSL binds tightly to PL mainly through hydrophobic interactions, van der Waals contacts, and a stable hydrogen-bonding network (Figure 4D). In the WFE–PL complex, Phe78, Ile79, Tyr115, Ala179, Pro181, and Phe216 showed relatively high favorable energy contributions (Figure 4B), and the dominant interactions comprised hydrophobic and van der Waals contacts as well as π–π stacking (Figure 4E). In the FGF–PL complex, Phe78, Ile79, Tyr115, Pro181, Phe216, and Arg257 contributed to binding (Figure 4C), with hydrophobic and van der Waals interactions accompanied by a limited number of hydrogen bonds as the main interaction types (Figure 4F). However, compared with GFSL, both WFE and FGF showed weaker interactions with these key residues, suggesting that GFSL more effectively exploits hotspot residues within the binding pocket to achieve stable association. Notably, residue decomposition also indicated that the same residue may exhibit different, or even opposite, energetic contributions when binding different peptides. For example, Phe78 contributed unfavorably in the GFSL–PL system, but favourably in the WFE–PL and FGF–PL systems. This discrepancy may be attributed to peptide-dependent differences in binding orientation and conformation within the pocket, leading to steric hindrance or conformational repulsion in the GFSL-bound state, whereas in the WFE- and FGF-bound states, Phe78 may adopt a geometry that favours hydrophobic contacts.

**Figure 5:**
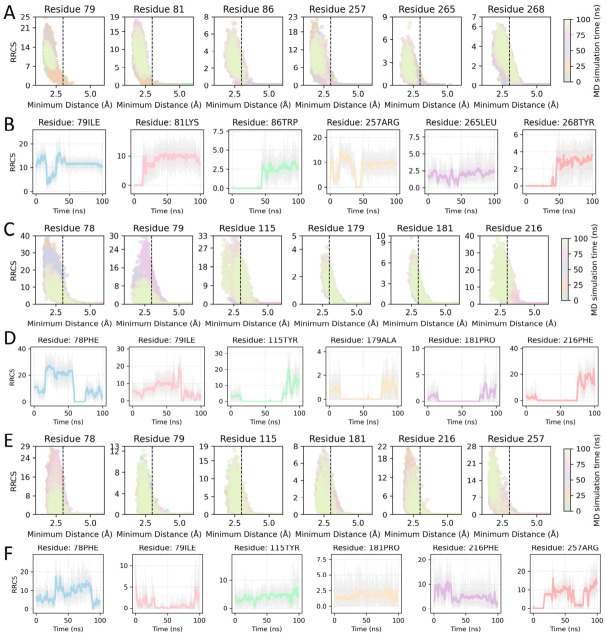
RRCS analysis of peptide–PL complexes: (**A**) Scatter plot of RRCS versus minimum distance for GFSL. (**B**) Time evolution of RRCS during MD simulation for GFSL. (**C**) Scatter plot of RRCS versus minimum distance for WFE. (**D**) Time evolution of RRCS during MD simulation for WFE. (**E**) Scatter plot of RRCS versus minimum distance for FGF. (**F**) Time evolution of RRCS during MD simulation for FGF.

To further assess the stability of peptide–PL binding from a dynamic perspective, RRCS analysis was performed to characterize the contact features between the peptides and key residues throughout the simulations. The results showed that GFSL maintained shorter contact distances and longer contact durations with the identified hotspot residues (Figure 5A,B), and these contacts gradually stabilized during the second half of the trajectory (after ~50 ns), indicating that GFSL adopts a tighter and more persistent binding mode with PL. In contrast, WFE exhibited larger fluctuations in contacts with key residues and formed contacts for shorter periods (Figure 5C,D), whereas FGF displayed the fewest persistent contacts with the key residues, consistent with an overall looser association (Figure 5E,F).

Taken together, the MM/PBSA binding free-energy calculations, residue-level energy decomposition, and RRCS-based dynamic contact analyses suggest that GFSL, WFE, and FGF can all interact favorably with PL, although they differ in binding strength, hotspot-residue usage, and dynamic interaction patterns. The comparatively better PL inhibitory activity of GFSL is likely attributable to its more favorable binding free energy and its more stable and persistent contacts with key residues during the simulations. This synergy between strong binding affinity and sustained hotspot engagement provides a molecular-level rationale for why GFSL outperforms WFE and FGF in inhibiting PL.

## 4. Conclusions

This study showed that enzymatic hydrolysis is an effective approach for improving the functional properties of pea protein and generating hydrolysates with both PL inhibitory activity and antioxidant capacity. Under optimized conditions, the resulting hydrolysate exhibited PL inhibitory activity together with ABTS^+^· and DPPH· radical scavenging capacities. Combined peptidomics, virtual screening, and in vitro validation identified three active PL-inhibitory peptides, GFSL, WFE, and FGF, among which GFSL displayed the highest activity. Molecular simulation indicated that peptide binding in the catalytic pocket of PL was primarily driven by hydrophobic interactions, van der Waals forces, and hydrogen bonding, with GFSL showing the most favorable binding mode.

Taken together, these results highlight PPH and derived peptides as promising natural ingredients for functional foods aimed at modulating lipid digestion. Further research is required to clarify peptide stability, gastrointestinal fate, and bioavailability before practical application.

## Figures and Tables

**Table 1:** Virtual screening, in vitro activity validation and MM/PBSA results.

Sequence	PeptideRanker	ToxinPred Prediction	Vina Score (kcal/mol)	IC_50_	MM/PBSA
LFL	0.944	Non-Toxin	−8.605	>1 mM	
LFE	0.509	Non-Toxin	−8.303	>1 mM	
GFSL	0.844	Non-Toxin	−8.814	337.81 ± 17.32 μM	−26.24 ± 5.25 kcal/mol
FGF	0.997	Non-Toxin	−9.525	689.45 ± 39.32 μM	−17.37 ± 3.20 kcal/mol
WFE	0.943	Non-Toxin	−9.577	473.32 ± 19.61 μM	−22.92 ± 3.43 kcal/mol

## Data Availability

The original contributions presented in this study are included in the article/Supplementary Materials. Further inquiries can be directed to the corresponding author. The LC-MS/MS proteomics data are publicly available via the ProteomeXchange Consortium (https://proteomecentral.proteomexchange.org, accessed on 25 March 2026) through the iProX partner repository under the dataset identifier PXD076130.

## Supplementary Materials

The following supporting information can be downloaded at https://www.mdpi.com/article/10.3390/foods15091523/s1. Table S1: Hydrolysis conditions of different enzymes. Table S2. Factor levels for the response surface design. Table S3. ANOVA results for the RSM model. Table S4. Peptide sequences identified in PPH.

## Abbreviations

Abbreviation	Full term
ABTS	2,2′-azinobis-(3-ethylbenzothiazoline-6-sulfonic acid)
ANOVA	analysis of variance
ANS	8-anilino-1-naphthalenesulfonic acid
CCD	central composite design
DCCM	dynamic cross-correlation matrix
DPPH	2,2-diphenyl-1-picrylhydrazyl
E/S	enzyme-to-substrate ratio
FEL	free energy landscape
GROMACS	GROningen MAchine for Chemical Simulations
IC_50_	half maximal inhibitory concentration
LC-MS/MS	liquid chromatography-tandem mass spectrometry
MD	molecular dynamics
MM/PBSA	molecular mechanics/Poisson–Boltzmann surface area
PCA	principal component analysis
PBS	phosphate-buffered saline
pNPB	p-nitrophenyl butyrate
PL	pancreatic lipase
PPH	pea protein hydrolysate
PPI	pea protein isolate
RMSD	root mean square deviation
RRCS	residue–residue contact score
RSM	response surface methodology
VMD	visual molecular dynamics

## References

[1] Ahmed S.K., Mohammed R.A. (2025). Obesity: Prevalence, causes, consequences, management, preventive strategies and future research directions. Metab. Open.

[2] Murphy N., Jenab M., Gunter M.J. (2018). Adiposity and gastrointestinal cancers: Epidemiology, mechanisms and future directions. Nat. Rev. Gastroenterol. Hepatol..

[3] Wu Z., Xia F., Wang W., Zhang K., Fan M., Lin R. (2025). The Global Burden of Disease Attributable to High Body Mass Index in 204 Countries and Territories from 1990 to 2021 with Projections to 2050: An Analysis of the Global Burden of Disease Study 2021. Eur. J. Heart Fail..

[4] Perdomo C.M., Cohen R.V., Sumithran P., Clément K., Frühbeck G. (2023). Contemporary medical, device, and surgical therapies for obesity in adults. Lancet.

[5] Mohsin N.U.A., Ahmad M., Farrukh M., Rafique S. (2025). Pancreatic lipase inhibitors as anti-obesity agents: A review of recent chemical scaffolds and their pancreatic lipase inhibitory potential. Med. Chem. Res..

[6] Liu T.-T., Liu X.-T., Chen Q.-X., Shi Y. (2020). Lipase Inhibitors for Obesity: A Review. Biomed. Pharmacother..

[7] Suryaningtyas I.T., Je J.-Y. (2023). Bioactive peptides from food proteins as potential anti-obesity agents: Mechanisms of action and future perspectives. Trends Food Sci. Technol..

[8] Wang X., Ai X., Zhu Z., Zhang M., Pan F., Yang Z., Wang O., Zhao L., Zhao L. (2022). Pancreatic lipase inhibitory effects of peptides derived from sesame proteins: In silico and in vitro analyses. Int. J. Biol. Macromol..

[9] Gan Y., Xie N., Zhang D. (2025). Pea-Derived Antioxidant Peptides: Applications, Bioactivities, and Mechanisms in Oxidative Stress Management. Chemistry.

[10] Hadidi M., Boostani S., Jafari S.M. (2022). Pea proteins as emerging biopolymers for the emulsification and encapsulation of food bioactives. Food Hydrocoll..

[11] Olsmats E., Rennie A.R. (2024). Pea protein [*Pisum sativum*] as stabilizer for oil/water emulsions. Adv. Colloid Interface Sci..

[12] Huang M., Tian M., Tan C., Ying R., Ahmad M., Hao G., Liao Q. (2024). Thermal stability, antioxidant activity and bioavailability of pea protein-naringin Pickering emulsion for enhanced delivery applications. Food Res. Int..

[13] Wu D.T., Li W.X., Wan J.J., Hu Y.C., Gan R.Y., Zou L. (2023). A Comprehensive Review of Pea (*Pisum sativum* L.): Chemical Composition, Processing, Health Benefits, and Food Applications. Foods.

[14] Parveen B., Rajinikanth V., Narayanan M. (2025). Natural plant antioxidants for food preservation and emerging trends in nutraceutical applications. Discov. Appl. Sci..

[15] Nasri M., Toldrá F. (2017). Chapter Four—Protein Hydrolysates and Biopeptides: Production, Biological Activities, and Applications in Foods and Health Benefits. A Review. Advances in Food and Nutrition Research.

[16] Czelej M., Garbacz K., Czernecki T., Wawrzykowski J., Waśko A. (2022). Protein Hydrolysates Derived from Animals and Plants—A Review of Production Methods and Antioxidant Activity. Foods.

[17] Lenth R.V. (2009). Response-Surface Methods in R, Using rsm. J. Stat. Softw..

[18] Ma J., Chen T., Wu S., Yang C., Bai M., Shu K., Li K., Zhang G., Jin Z., He F. (2019). iProX: An integrated proteome resource. Nucleic Acids Res..

[19] Chen T., Ma J., Liu Y., Chen Z., Xiao N., Lu Y., Fu Y., Yang C., Li M., Wu S. (2022). iProX in 2021: Connecting proteomics data sharing with big data. Nucleic Acids Res..

[20] Rathore A.S., Choudhury S., Arora A., Tijare P., Raghava G.P.S. (2024). ToxinPred 3.0: An improved method for predicting the toxicity of peptides. Comput. Biol. Med..

[21] Eberhardt J., Santos-Martins D., Tillack A.F., Forli S. (2021). AutoDock Vina 1.2.0: New Docking Methods, Expanded Force Field, and Python Bindings. J. Chem. Inf. Model..

[22] Schrödinger, Inc. (2023). The PyMOL Molecular Graphics System, Version 2.6.0.

[23] Salentin S., Schreiber S., Haupt V.J., Adasme M.F., Schroeder M. (2015). PLIP: Fully automated protein-ligand interaction profiler. Nucleic Acids Res..

[24] Humphrey W., Dalke A., Schulten K. (1996). VMD: Visual molecular dynamics. J. Mol. Graph..

[25] Grant B.J., Rodrigues A.P., ElSawy K.M., McCammon J.A., Caves L.S. (2006). Bio3d: An R package for the comparative analysis of protein structures. Bioinformatics.

[26] Valdés-Tresanco M.S., Valdés-Tresanco M.E., Valiente P.A., Moreno E. (2021). gmx_MMPBSA: A New Tool to Perform End-State Free Energy Calculations with GROMACS. J. Chem. Theory Comput..

[27] Bouysset C., Fiorucci S. (2021). ProLIF: A library to encode molecular interactions as fingerprints. J. Cheminformatics.

[28] Han W., Chen Z., Wang M.W., Zhou Q. (2025). gmx_RRCS: A Precision Tool for Detecting Subtle Conformational Dynamics in Molecular Simulations. J. Mol. Biol..

[29] R Core Team (2023). R: A Language and Environment for Statistical Computing.

