# Supplementary material for: Mechanistic Insights into Pancreatic Lipase Inhibition by Pea-Derived Peptides: Integrating Process Optimization, Activity Assays, Docking, and Molecular Dynamics
Source: Foods. 2026 Apr 28;15(9):1523. doi: 10.3390/foods15091523 (PMC13163611; doi:10.3390/foods15091523)
Supplement: Supplementary file 1 [file foods-15-01523-s001.zip › foods-4244493-supplementary.pdf]

Supporting Information:

Identification and Mechanistic Characterization of Pancreatic Lipase-Inhibitory Peptides from Pea Protein Hydrolysates by Peptidomics, Molecular Docking, and Molecular Dynamics Simulation

Yi Zhao, Jinhong Wang, Xiang Li, Guizhao Liang *

Key Laboratory of Biorheological Science and Technology, Ministry of Education, Bioengineering College, Chongqing University, Chongqing 400044, China

*Corresponding Author:

Dr. Guizhao Liang

Chongqing, China.

Tel.: 023-65102507

E-mail: gzliang@cqu.edu.cn

Table S1. Hydrolysis conditions of different enzymes.

Type	pH	Temperature（℃）
Trypsin	8	37
Pepsin	2.5	37
Papain	7	50
Alkaline protease	7	50
Neutral protease	10	50

Table S2. Factor levels for the response surface design.

Std	Run	A	B	C	D	Inhibition
23	1	10.5	45	3	3000	27.9897
9	2	10	40	2	6000	26.4875
3	3	10	50	2	4000	24.0074
11	4	10	50	2	6000	21.1431
12	5	11	50	2	6000	28.2613
14	6	11	40	4	6000	27.4194
15	7	10	50	4	6000	32.4584
24	8	10.5	45	3	7000	31.2683
28	9	10.5	45	3	5000	43.8836
16	10	11	50	4	6000	25.0993
18	11	11.5	45	3	5000	23.5716
6	12	11	40	4	4000	22.8122
25	13	10.5	45	3	5000	43.4882
8	14	11	50	4	4000	24.2052
13	15	10	40	4	6000	32.9649
22	16	10.5	45	5	5000	30.4585
26	17	10.5	45	3	5000	41.1969
29	18	10.5	45	3	5000	41.495
4	19	11	50	2	4000	26.6224
17	20	9.5	45	3	5000	26.4585
5	21	10	40	4	4000	32.8089
19	22	10.5	35	3	5000	33.9971
30	23	10.5	45	3	5000	42.5323
1	24	10	40	2	4000	27.8925
21	25	10.5	45	1	5000	29.1127
2	26	11	40	2	4000	32.5883
7	27	10	50	4	4000	31.1621
20	28	10.5	55	3	5000	25.5618
10	29	11	40	2	6000	33.497
27	30	10.5	45	3	5000	41.1796

Table S3. ANOVA results for the RSM model.

Source	Degree of Freedom	Sum of squares	Mean of squares	*F* value	*P* value
FO(A, B, C, D)	4	87.2859	21.8215	19.2739	0.0000
TWI(A, B, C, D)	6	196.0741	32.6790	28.8638	0.0000
PQ(A, B, C, D)	4	981.2339	245.3085	216.6695	0.0000
Lack of fit	10	9.8890	0.9889	0.6970	0.7073
Pure error	5	7.0936	1.4187		
Residuals	15	16.9827	1.1322		

FO: first order; TWI: two-way interactions; PQ: pure quadratic

Table S4. Peptide sequences identified in PPH.

Sequence	Length	Sequence	Length	Sequence	Length	Sequence	Length
LLF	3	LNF	3	LFLQ	4	LLSL	4
WRL	3	VAW	3	VEFL	4	FDLL	4
VVF	3	LVY	3	KLLL	4	DLLR	4
FEK	3	LVR	3	LLGF	4	LKLF	4
LYL	3	MVL	3	VLLV	4	LLAL	4
FVF	3	QYL	3	PLLR	4	YLEL	4
SLL	3	NPR	3	VVAL	4	TFVL	4
LLL	3	ALW	3	LEVL	4	LLVQ	4
QLL	3	SLW	3	DLRL	4	LANL	4
LFL	3	FML	3	VTLR	4	YDAL	4
YLF	3	WKS	3	VGSL	4	FLLR	4
FAL	3	PKL	3	DFTF	4	VVLE	4
DLL	3	YML	3	LNRF	4	NYGL	4
TLF	3	FRL	3	LEKL	4	VTEL	4
YSF	3	GLR	3	LYRF	4	PLRL	4
VRL	3	PVL	3	LEFL	4	YKLL	4
GLL	3	LPH	3	FDPF	4	FNLR	4
NLF	3	DLR	3	KLFL	4	VTAL	4
SYL	3	KGF	3	LRLL	4	HLLL	4
LFE	3	VSW	3	VALL	4	LAPL	4
LSF	3	ELL	3	RNPF	4	YLFK	4
LRL	3	WLR	3	SGLL	4	DLGL	4
YVL	3	FGF	3	YLFR	4	VEKL	4
ELF	3	LFW	3	LVKL	4	LHML	4
LKL	3	VLW	3	KFLL	4	LFLK	4
FFK	3	WLE	3	LLLL	4	VVFK	4
EFL	3	WFE	3	LLLR	4	LKPF	4
VDL	3	VMF	3	FLVR	4	LAAL	4
LQL	3	VFK	3	VRGL	4	AVAL	4
VKL	3	LKF	3	LVLL	4	FLGR	4

LVF	3	VHP	3	FFSN	4	LFLE	4
VLR	3	ELY	3	LHVL	4	NLFF	4
VFW	3	LDF	3	FLLF	4	RLLY	4
VYL	3	MGF	3	VLTL	4	LLKF	4
LWL	3	LPF	3	LLRL	4	VLVK	4
YLL	3	LWR	3	LFSY	4	FSLR	4
VDF	3	QLK	3	TALL	4	LDRQ	4
LDL	3	FSL	3	VLLQ	4	FLVM	4
LNL	3	HGL	3	LYPL	4	LKLY	4
WAL	3	LQY	3	VKLL	4	VGHS	4
LHL	3	LPR	3	VLKL	4	KLLR	4
YAL	3	LSY	3	LHPG	4	EFLR	4
LGR	3	LKA	3	LKVL	4	RKRL	4
VAR	3	LAW	3	LLEY	4	VFKK	4
FLM	3	PNK	3	SPPL	4	PATL	4
TFL	3	YLM	3	GRLF	4	LRDLL	5
FRF	3	YPL	3	GKLF	4	LALTL	5
VVK	3	PLY	3	LLFL	4	KDLLL	5
DRL	3	LEY	3	LEAL	4	DLRLL	5
FLK	3	LMY	3	SSLL	4	LFLQL	5
FLY	3	GEL	3	YLPF	4	RKFLL	5
LLR	3	TLP	3	LKYL	4	EFDLR	5
KFL	3	QNL	3	LLNL	4	VGSLL	5
FLA	3	KFT	3	YALL	4	TLLFL	5
GWL	3	ELK	3	GFSL	4	VRDLL	5
LQF	3	FSK	3	VHLF	4	VADLL	5
LLK	3	NHV	3	LKFL	4	LAVFK	5
WLL	3	NKE	3	VAWR	4	LVKLL	5
RVL	3	TFR	3	TLFE	4	KLLRL	5
SKW	3	LFR	3	VADL	4	LLRNL	5
KPF	3	RLL	3	NFPF	4	LLRLL	5
KLL	3	LKW	3	LRLV	4	LFSLF	5
FKF	3	PNR	3	SKVV	4	FKKLF	5
YMF	3	LALF	4	FDLR	4	LKFLT	5
